# Importance of Saprotrophic Freshwater Fungi for Pollen Degradation

**DOI:** 10.1371/journal.pone.0094643

**Published:** 2014-04-14

**Authors:** Christian Wurzbacher, Stefan Rösel, Anna Rychła, Hans-Peter Grossart

**Affiliations:** 1 Leibniz-Institute of Freshwater Ecology and Inland Fisheries, Department Experimental Limnology, Stechlin, Brandenburg, Germany; 2 Potsdam University, Institute for Biochemistry and Biology, Potsdam, Brandenburg, Germany; Leiden University, Netherlands

## Abstract

Fungi and bacteria are the major organic matter (OM) decomposers in aquatic ecosystems. While bacteria are regarded as primary mineralizers in the pelagic zone of lakes and oceans, fungi dominate OM decomposition in streams and wetlands. Recent findings indicate that fungal communities are also active in lakes, but little is known about their diversity and interactions with bacteria. Therefore, the decomposer niche overlap of saprotrophic fungi and bacteria was studied on pollen (as a seasonally recurring source of fine particulate OM) by performing microcosm experiments with three different lake types. Special emphasis was placed on analysis of fungal community composition and diversity. We hypothesized that (I) pollen select for small saprotrophic fungi and at the same time for typical particle-associated bacteria; (II) fungal communities form specific free-living and attached sub-communities in each lake type; (III) the ratio between fungi or bacteria on pollen is controlled by the lake's chemistry. Bacteria-to-fungi ratios were determined by quantitative PCR (qPCR), and bacterial and fungal diversity were studied by clone libraries and denaturing gradient gel electrophoresis (DGGE) fingerprints. A protease assay was used to identify functional differences between treatments. For generalization, systematic differences in bacteria-to-fungi ratios were analyzed with a dataset from the nearby Baltic Sea rivers. High abundances of *Chytridiomycota* as well as occurrences of *Cryptomycota* and yeast-like fungi confirm the decomposer niche overlap of saprotrophic fungi and bacteria on pollen. As hypothesized, microbial communities consistently differed between the lake types and exhibited functional differences. Bacteria-to-fungi ratios correlated well with parameters such as organic carbon and pH. The importance of dissolved organic carbon and nitrogen for bacteria-to-fungi ratios was supported by the Baltic Sea river dataset. Our findings highlight the fact that carbon-to-nitrogen ratios may also control fungal contributions to OM decomposition in aquatic ecosystems.

## Introduction

The functional role of aquatic fungi and bacteria in decomposing vascular plant litter in streams and marshlands has been assessed frequently (reviewed in [1]). Higher filamentous aquatic fungi (aquatic hyphomycetes) are regarded as major decomposers of large vascular plant remnants in aquatic ecosystems, often with more than 95% dominance over contributing bacteria [2], [3]. However, in lentic systems, algae are the primary producers and aquatic bacteria trigger internal mineralization in the pelagic zone of lakes by their metabolic capacity for carbon and nutrient uptake (e.g., [4]). They metabolize rapidly dissolved organic matter (DOM) released by photoautotrophic phytoplankton and/or microbial hydrolysis of organic aggregates [5], [6]. Fungi do exist as small zoosporic (lower) fungi known to parasitize on planktonic organisms (e.g., [7]–[9]). While it has been noted that lower fungi can also act as primary decomposers (listed by substrates in [10]) this has been rarely considered relevant until recently [11]–[13]. Therefore, little is known about fungal contributions to the degradation of fine particulate organic matter (FPOM) derived from algae, zooplankton, shredder activities, or other sources in the pelagic zone. Additionally, new aquatic fungal lineages are steadily uncovered by molecular tools (e.g., [14]–[18]). The ecology of these lineages is mostly unknown and highlights the need to reconsider the ecological role of fungal organisms in freshwater ecosystems.

Our aim was to investigate the saprotrophic fungal community in lakes and to highlight their occurrence on a frequent and important high-quality FPOM source in temperate and boreal lakes, i.e. wind-distributed pollen. The broad boreal distribution of pine trees and its importance in forestry renders pine pollen a frequent and quantitatively important source of terrestrial subsidies in many freshwater systems, particularly in the northern hemisphere [19], [20]. This “pollen rain” can account for a substantial fraction of allochthonous and atmospheric nutrient deposition into oligotrophic lakes [19], [21]–[23]. For instance, [20] estimated that almost half of the annual atmospheric phosphorous load of oligotrophic Lake Stechlin (Germany) is derived from pollen and occurs in a very concise timeframe.

Pollen grains are in the size range of algae and planktonic aggregates (ca. 50 µm), and establishment of hyphal networks might be retarded due to their limited surface size. It has been already shown that the fungal biomass on POM in streams measured by ergosterol decreased with decreasing POM size class, which suggests a bacterial predominance on smaller particles [24]. However, ergosterol-based biomass estimations are problematic for lower fungal phyla, and measured differences will account only for the dynamics of hyphomycetes. As a consequence, this has often led to a one-sided perspective of either lower or higher fungi, and only a few studies targeted lower and higher fungi simultaneously [12], [25], [26]. As an alternative to the previously mentioned retarded development of fungal biomass on small particles such as pollen, a shift in fungal phyla and/or morphologies towards small unicellular yeast-like and zoosporic fungi is likely (morphotype concept; [27]). Therefore, we expect that pollen grains will structure the fungal community accordingly. It is already known that a diverse range of fungi is frequently associated with pollen [12], [28]–[30], and many, but not all, of them can be regarded as zoosporic fungi [12], [29]. A second aspect addresses the lake specificity of microbial communities. Whereas it is known that bacterial community composition shows lake-specific patterns [31], [32], almost nothing is known about the lake-specificity of saprotrophic fungal communities. Therefore, it is necessary to analyze the fungal community in detail, i.e., to simultaneously examine the occurrence of lower and higher fungi on pollen grains, reveal their lake-specificity, and compare fungal patterns with those of bacterial communities.

Saprotrophic fungi and saprotrophic bacteria seem to have a pronounced niche overlap, and both depend strongly on the organic carbon content across biomes (r^2^ = 0.91, [33]). In general, multiple interrelationships have been described between the two microbial groups. Exploitation competition has been described as the dominant relationship [34], in which substrates are potentially apportioned by molecule classes [35] through differential enzyme expression [36]. Habitat-specific substrate quantity [37] and, in particular, substrate quality, i.e. carbon source, C∶N, or N∶P ratio, are important determinants for shifts of the bacterial∶fungi (B∶F) ratio [24], [38], [39]. Likewise, the soluble parameters DOM, dissolved organic carbon (DOC), and pH have been assigned to major changes in biomass of aquatic fungi or bacteria [40]–[44].

To account for systematic differences between the dominance of fungi and bacteria related to environmental parameters, their ratio (B∶F) was used as a metric to characterize soil ecosystems [45]. Both C∶N and pH are important factors for local microbial ratios [33], [46] and communities [47]–[49].

To test for the importance of aquatic fungi on small FPOM, we performed a laboratory microcosm experiment with pollen grains and studied their diversity, community structure, and the decomposer niche overlap with bacteria on pollen using water from three lake types differing in refractory DOC and pH. We hypothesized:

1. that small unicellular and zoosporic aquatic fungi will greatly benefit from input of pollen, in particular saprotrophic fungi;
2. that lake-specific communities of microbes are well adapted to pollen following a progressive succession;
3. that different lake properties define systematic differences between the dominance of fungi and bacteria and therefore shape the niche overlap between both saprophytic microbial groups.

## Material and Methods

### Experimental setup

**Figure 1:**
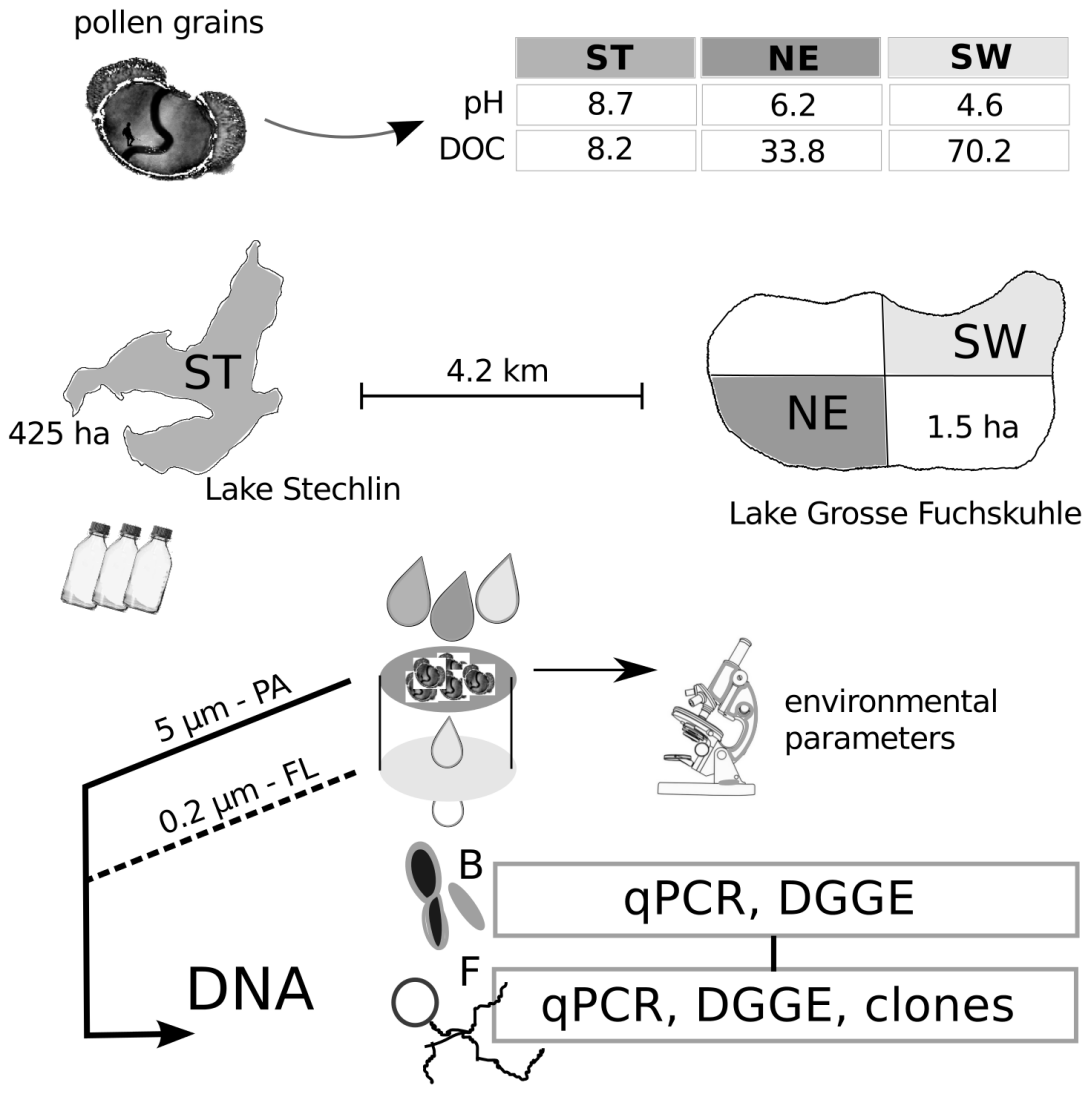
Experimental setup. Experimental setup showing origin, distance, and features of the three lakes: Lake Stechlin (ST) and Lake Grosse Fuchskuhle Northeast (NE) and Southwest basins (SW). Pollen incubations were sampled four times within two weeks for molecular analyses (DGGE, qPCR, clone libraries), chemical side parameters (see main text), and microscopy.

The experimental setup and lake types (treatments) were described in detail by [20]. In brief, pollen of *P. sylvestris* was collected during the pollen season on a dry and canopied surface close to the shore of Lake Stechlin in late May 2008. The water samples were taken at 16^th^ of June 2008, which was also the start of the experiment. Sampled lakes are part of the nature conservation park “Stechlin” in Northeast Germany (permission for sampling was granted by state edict 791-7df, Brandenburg, Germany). Briefly, 100 µm gauze pre-filtered water samples were obtained from oligotrophic Lake Stechlin (ST: *pH = 8.2, DOC = 8.2 mg l^−1^*) and from the eutrophic northeast basin (NE: *pH = 6.5, DOC = 33.8 mg l^−1^*) and the dystrophic southwest basin (SW: *pH = 4.8, DOC = 70.2 mg l^−1^*) of Lake Grosse Fuchskuhle. These two basins are considered as two lake systems with distinct microbial communities [31], [32]. The DOC consists mainly of humic matter, which is responsible for the light and dark brown watercolor of the two basins. Following collection, the samples were incubated in triplicate for 16 days in the dark at 18°C with 50 mg l^−1^ of 2–3 weeks old pollen of *Pinus sylvestris* (freshness/viability was confirmed in the microscope by observing the formation of pollen tubes: 21% of pollen grains in NE water developed tubes) in a total volume of 2 liters (Figure 1). Samples of 200 ml each were taken on days 1, 2, 3, 5, 10, and 16 of the experiment and samples from day 1 and 3 were taken for chemical measurements only, and hence were given elsewhere [20]. An initial sample at day 0 was taken before pollen addition. Until the last sampling date the total incubation volume was reduced to approximately 0.8 l (i.e. by 60% of the initial volume).

All samples were subdivided to measure side variables (120 ml): total organic carbon (TOC), DOC, particulate organic carbon (POC), soluble reactive phosphorus (SRP), total dissolved phosphorus (TDP), dissolved organic phosphorus (DOP), particulate phosphorous (PP), particulate nitrogen (PN), and total dissolved nitrogen (TDN; for more details and biogeochemical results, see [20]). In addition, DNA was acquired for phylogenetic analyses of microbes (fungi and bacteria) in two size fractions. The latter were obtained by filtering 40 ml of each sample by vacuum filtration in two subsequent steps over a 5.0 µm filter and a 0.2 µm filter (polycarbonate, Millipore). Both filters were stored at −20°C until DNA extraction. The fractionated filtration allowed for further discrimination between particle-associated (PA) and free-living (FL) microbes, respectively (Figure 1). Fungal cell walls were stained with Calcofluor White as described previously [20], [50]. The most relevant processes during the pollen decomposition of this setup can be summarized as follows (presented in [20]): (i) all three systems switched from a nutrient limitation to a carbon limitation characterized by steadily increasing concentrations of dissolved nitrogen and phosphorous; (ii) pollen rapidly decomposed within two weeks; (iii) no significant differences in pollen decomposition and nutrient release between the lake systems were found; (iv) pollen leachate stimulated bacterial growth in the very beginning, whereas (v) fungi emerged after a lag phase.

Based on these observations, we defined days 2 and 5 as the early phase characterized by strong changes in microbial biomass and chemistry and days 10 and 16 as the late phase of decomposition with rather stable conditions for the above-mentioned parameters (Table S1). In addition, the initial C∶N POM values in the water before pollen addition were comparable across all treatments (28.9 [+/−1.8] by mass).

### Aminopeptidase activity measurements

We exemplarily measured the leucine-aminopeptidase activity at day 3 when bacteria were dominant. Samples that had been incubated for three days without any pollen addition served as controls. Fluorogenic leucine-MCA (Sigma) was added at saturating concentration (0.1 mM) to a 4 ml subsample as a substrate analogue. After 1 h of incubation at 18°C, pH was adjusted to pH>10 using borate buffer directly before fluorescence measurements [51]. Relative fluorescence values were determined with a fluorometer at λ_ex_ = 365 nm and λ_em_ = 440 nm (Kontron SFM-25). The humic-matter-rich water from SW had a weak quenching effect, which we adjusted for by calibrating each treatment with MCA (Sigma).

### DNA extraction

Stored filters were extracted with a peqGOLD Tissue DNA Kit (PEQLAB, Germany) with an additional upstream mechanical disruption step in which the filters were added to 0.25 g of 0.1 mm zirconium beads and mechanically disrupted by bead beating in a benchtop mill (Retsch MMX 400, f = 30 s^−1^, 2 min). DNA concentrations were measured via PicoGreen (Invitrogen) using a microplate reader (Synergy 2, BioTek).

### Primer design for aquatic fungi

**Figure 2:**
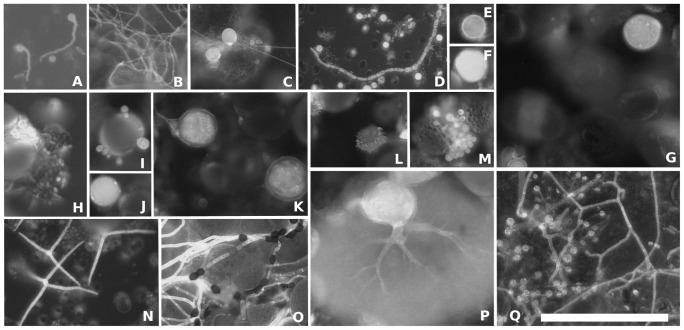
Examples of fungal morphologies. Examples of fungal morphologies observed during the pollen degradation experiment, stained by Calcofluor White. Sporangia belonging to *Chytridiomycota*, filamentous fungi, yeast-like growth forms, and conidia of aquatic hyphomycetes are documented. (A) germinating zoospores. (B, C, D, H, O, Q) different kinds of observed fungal hyphae without clamps, partly melanized (H and O). (D) small, yeast-like growth forms. (E) potential resting spore (chytrid). (F) spore or sporangium. (C, G, I, J, L, M, P, Q) sporangia of different species of *Chytridiomycota*, where (J) resembles a typical *Rhizophydium* sporangium and (P) resembles *Chytridiomycetes*. (K) spore attached to filaments resembles a morphology similar to species of *Glomeromycota* or *Zygomycetes*. (N) typical tetraradiate conidia of an aquatic hyphomycete. (I, Q) mycoparasites of sporangia and fungal filaments. Bar = 100 µm.

Microscopic observations (Figure 2) confirmed the presence of basal zoosporic and higher filamentous fungi. Therefore, a primer system was needed to balance these two groups. To accomplish this, we evaluated the usage of different established and new primer sets mainly targeting the ribosomal large subunit (LSU) region. The primer sets were different DGGE sets targeting the LSU (three sets), the internal transcribed spacer (ITS) (one set), and the small subunit (SSU) (one set), as well as two primer sets for qPCR and a single primer set for clone libraries spanning regions D1–D8 of the LSU. The sequences and DGGEs presented in the main text are explicitly based on results obtained for the LSU region (see Material S1 for results on SSU and ITS). New primers were designed using ARB [52] and the SILVA database 108 [53] for ribosomal LSU sequences to cover most fungi including the majority of lower aquatic fungi. For DGGE, we used a newly developed primer pair LR78 (AGATCTTGGTGGTAGTAGCAA) modified from LR7 [54] and RD78-GC (GC-clamp-TGTTTTAATTAGACAGTCAGATTC); for clone libraries we used NL1a (AAGCATATCAATAAGCGGAGG), modified from [55] as a forward primer and RD78 (without GC-clamp) as reverse primer; and for qPCR we used our new primer pair QRTfungf (CCTTAGACTGACAGATTAA) and QRTfungr (GTTTGATCTCAGTTCGAG). Detailed information on primer design, evaluation, regions, and handling is given in the supporting information (Material S1).

### DGGE

DGGE was performed following [56]. Briefly, PCR products with a GC clamp were separated in a polyacrylamide gel (7%) with a denaturing gradient of urea and formamide. Gels were stained with SybrGold (Molecular Probes), and distinct bands were excised under low-UV light for reamplification at the same cycling conditions as initial PCRs. Resulting PCR products were Sanger-sequenced. We used a 50 µl reaction mix (Crimson Taq, New England Biolabs, 2.5 mM MgCl_2_) with the following cycling conditions for fungal-specific primer pair LR78/RD78: 33 cycles of 95°C for 1 min and 2.5 min at 60°C annealing/elongation. Fungal PCR products were subsequently separated in a denaturing gradient of 30–45%. For bacterial DGGEs, we utilized primer pair 341f-GC and 907r with DGGE conditions as described in [31].

### Clone libraries

Clone libraries were constructed solely for fungi. Approximately 2000 nt of the LSU rRNA gene were amplified, spanning regions D1 to D8, using primer NL1a and RD78 with the following cycling conditions: 95°C for 3 min; 35 cycles at 95°C for 1 min and 60°C for 4 min; and a final elongation step for 30 min at 68°C. Obtained PCR products were quality checked and purified with silica columns (Qiagen PCR purification). For phylogenetic comparison of lake types (treatments), PCR products derived from the DNA of both size fractions were pooled together. Similarly, all those pools derived from the two early sampling dates (days 2 and 5), and the two from the late sampling dates (days 10 and 16) of one replicate per treatment were merged, which resulted in six pools. These were cloned into pGEM-T Easy (Promega) vectors and transformed in *Escherichia coli.* To preselect clones for sequencing, we screened at least 96 clones per library and digested the insert with the enzymes MSPI and RsaI (NEB; 0.2 units each). The obtained banding pattern was analyzed on a 3% agarose gel stained with SybrGold (Molecular Probes, 1∶10000). Representative clones of inserts with potentially different restriction fragment length polymorphism (RFLP) patterns were selected and their inserts were sequenced with primers T7, SP6, and NL4Seq (TTGAAACACGGACCAAGGA). Two additional clone libraries were prepared to compare DNA for both size fractions (PA and FL) without pre-selection. For this, we used DNA from the ST treatment at day 5.

### Phylogenetic analysis

All full-length sequences of our clone libraries (>1.8 knt) were aligned using the SINA web aligner (V1.2.9; [57]) and integrated into the SILVA LSU reference database (108) using ARB. Sequences were screened against the SILVA LSU database for potential PCR artifacts using UCHIME [58] and alignments were manually inspected for alignment errors before building a new reference database. For phylogenetic analysis, curated sequences were added to the base tree by quick-add parsimony using the LSU positional variability filter provided by SILVA. They were exported together with their potential next relatives to calculate a refined maximum likelihood subtree using FastTree 2.1 based on 1000 calculations choosing a GTR-model with a Gamma20 likelihood [59]. The resulting tree was re-imported into ARB. Partial sequences were derived from clones, and DGGE bands of LSU primer systems (see Material S1) were aligned against our new reference database by SINA and added to the phylogenetic tree using the quick-add parsimony option. Full-length sequences were submitted to EMBL under the following accession numbers: HE806142–HE806185. Shorter sequences (DGGE bands and partial clone sequences) are provided in the supporting information (Material S2).

### Quantitative real-time PCR (qPCR)

In order to investigate the biomass-dynamics of fungi and bacteria during pollen degradation, we followed molecular quantities of ribosomal gene copies. For qPCR, we used EvaGreen (BioRad) with a 25–500 pg template in a total volume of 13 µl; a CFX96 cycler (BioRad); our newly designed primer pair QRTfungf and QRTfungr to quantify fungal ribosomal copy numbers (cycling conditions: 98°C for 2 min followed by 40 cycles of 98°C for 10 sec, 60°C for 8 sec, and 65°C for 15 sec); and primer pair Ba519f and Ba907r for ribosomal bacterial genes (under conditions according to [60] and [61]). A purified and quantified PCR product (Qiagen PCR Purification Kit) served as the standard for a dilution series in 1∶10 dilution steps. Quality control was ensured using meltcurve analysis following amplification and agarose gel electrophoresis with randomly chosen qPCR reactions. Fungal copy numbers roughly followed the values of prevalence of infection from [20] (r = 0.67).

### Statistics

DGGE community profiles were transformed into a digital binary matrix with the GelCompar II software package (version 5.0, Applied Maths). Internal variation among replicates was resolved by cluster analysis based on Dice dissimilarities and UPGMA. This internal control assured sufficient similarity between the replicates (Bacteria: 89% [day 2] to 76% [day 16], Fungi: 62% [day 2] to 70% [day 16]) and allowed choosing a representative sample for time-series analysis (Figure S1). For fungi, one replicate each of Lake Stechlin (FL) and Lake Fuchskuhle SW (PA) did not cluster within the corresponding replicate group at day 16. For bacteria, previously identified chloroplast bands were excluded from community statistics. Time-series DGGE patterns were analyzed as non-metric multidimensional scaling (NMDS) ordination plots based on the Kulczyński dissimilarity index (vegan package, V 1.17-10; [62]; R (http://www.r-project.org/). Significant category separations (treatment and size fractions, respectively) were investigated at a 0.95 confidence level and were visualized by plotting the deviation from average using an ellipse based around centroids. To account for possible metric factors that influence the community structure presented in the NMDS, chemical parameters and time were fitted into the two-dimensional ordination as vectors (9999 permutations, stratified within size-fractions, p<0.01). The relationships between bacterial and fungal fingerprints were examined using a Mantel test [63] with 9999 randomized runs using Spearmańs rank correlation for each size fraction.

For qPCR data, a two-way analysis of variance (ANOVA) with lake type and time as well as size fraction and time as factors was applied. Data were ln-transformed if needed (Smirnov-Kolmogorov test), and Levene's test was used to ensure the homogeneity of variances. Similarly, differences in aminopeptidase activities were confirmed using one-way ANOVA with Tukey's HSD-Post-Hoc test (α<0.05).

The differences in average B∶F ratios between treatments were analyzed by a non-parametric Kruskal-Wallis test (K.–W.; >2 groups). Furthermore, the late phase of the experiment (approximating the climax community) allowed us to set the B∶F ratio in relation to external factors. Since we were operating in a closed system, we expected that the B∶F would in turn influence the dissolved parameters. Hence, we chose to calculate the Spearman's rank correlation between the two measures. In order to investigate a possible linearity with C∶N we ln-transformed the data and calculated the Pearson's correlation. In a continuative step and as a conclusion of the results (see the Results section), we tried to find a relationship between carbon, pH, nitrogen and phosphorous with B∶F in an environmental Baltic Sea dataset, and we reanalyzed the data obtained from [44] with general linear models to apply our assumptions on a larger scale. The authors measured the biomass of bacteria (cell counts × biovolume) and fungi (ergosterol) from 33 Baltic rivers (Baltic Sea catchment). We excluded rivers with missing pH values and the two highest B∶F values to avoid skewing (River Vistula and Mörrumsån) and finally covered a pH range of 5.9–8.7 and a DOC range of 3.5–19.6 mg l^−1^. Based on the assumption that a combination of environmental variables determines B∶F, we further computed the least absolute shrinkage and the selection operator (LASSO) with the least angle regression solution for possible predictors using the R package “lars” (http://cran.r-project.org/web/packages/lars/index.html) to select the most probable predictor [64]. For the subsequent general linear model, the normal distribution and independence of model residuals were confirmed by scatterplots. The achievement of significant improvement in the models by adding predictors was tested with classical ANOVA (DOC-model (r^2^ = 0.152, dF = 32) vs. null model: p = 0.024; DOC+DON vs. DOC-model: p = 0.038).

## Results and Discussion

### Microbial colonization of pollen grains by freshwater fungi and bacteria

**Figure 3:**
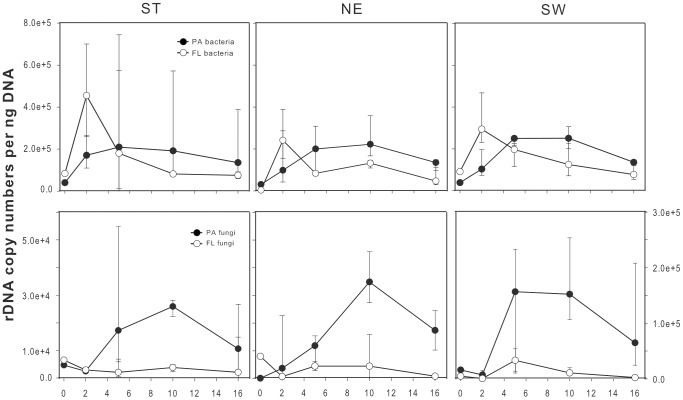
QPCR results showing fungal and bacterial succession on and surrounding pollen. Absolute copy numbers of bacterial and fungal ribosomal genes of treatments (ST = Lake Stechlin and Grosse Fuchskuhle basins: NE = Northeast, SW = Southwest) determined by qPCR for the course of the experiment. Size fractionation made it possible to split the results into two subcategories: free-living and particle associated microorganism. Median with standard deviation is shown (n = 3).

Pollen rapidly decomposed within 16 days of incubation. After pollen were exposed to lake water, pollen delivered an easily accessible soluble and particulate OM. Zoosporic fungi and filamentous forms emerged, which led to an aggregation of pollen grains [20]. The enzymatic activity of leucine-aminopeptidase was boosted by pollen in ST and NE, whereas it was negligible in the no-pollen controls and SW (day 3, Figure S2). The reduced activity in SW was due to the pH values, as it is known that this enzyme fails to work at low pH [65], [66]. In order to have a more detailed look at the microbial succession, we quantified the ribosomal gene abundance (Figure 3). The succession was comparable in all treatments. FL bacteria exhibited an early peak at day 2 and were paralleled by a smooth but steady increase in PA bacteria with maximal mean values between days 5 and 10. In contrast, FL fungal quantities stayed at a low level during the experiment in all treatments, while PA fungi increased continuously from day 5 on (Figure 3). Total fungal copy numbers differed significantly between treatments and size fractions (Table 1 & Table 2).

**Table 1:** Results of two-way ANOVA on ribosomal copy numbers of fungi and bacteria in particular lake types.

Microbial group	Lake type	Factor	Df_ef,err_	F	P
**Bacteria**	ST	Fraction	1,16	1.55	0.231
		Time	3,16	2.05	0.147
		Fraction × Time	3,16	3.07	0.058
	NE	Fraction	1,16	3.14	0.096
		Time	3,16	3.69	**0.034**
		Fraction × Time	3,16	2.76	0.076
	SW	Fraction	1,16	0.47	0.503
		Time	3,16	4.46	**0.019**
		Fraction × Time	3,16	7.04	**0.003**
**Fungi**	ST	Fraction	1,16	14.39	**0.002**
		Time	3,16	2.20	0.128
		Fraction × Time	3,16	1.96	0.160
	NE	Fraction	1,16	29.69	**0.000**
		Time	3,16	4.87	**0.014**
		Fraction × Time	3,16	2.55	0.092
	SW	Fraction	1,16	9.04	**0.008**
		Time	3,16	3.35	**0.045**
		Fraction × Time	3,16	1.99	0.156

Molecular quantities of bacteria and fungi of a lake type depending on factors: fraction, sampling date (Time) and the interaction (Fraction × Time). Df – degrees of freedom between and within groups, F – values of the F – test, P – probabilities values; ST – alkaline Lake Stechlin, NE – neutral northeast and SW – acidic southwest basin of Lake Grosse Fuchskuhle.

**Table 2:** Results of two-way ANOVA on total molecular quantities of bacteria and fungi.

Microbial group	Fraction	Factor	Df_ef,err_	F	P
**Bacteria**	Total	Treatment	2,24	2.30	0.122
		Time	3,24	4.97	**0.008**
		Treat × Time	6,24	0.41	0.863
**Fungi**	Total	Treatment	2,24	10.43	**0.001**
		Time	3,24	10.78	**0.000**
		Treat × Time	6,24	0.85	0.544

Molecular quantities of bacteria and fungi depending on lake type (Treatment), sampling date (Time) and the interaction (Treat × Time). Df – degrees of freedom between and within groups, F – values of the F – test, P – probabilities values.

Our qPCR data confirm earlier observations: free-living bacteria exhibited an early maximum, whereas fungi appeared after a lag phase (estimated then by number of sporangia per pollen grain) [20]. It is clear that fungi are restricted to particles, which confirms their strong assignment to surfaces, and that bacteria use the same surfaces in considerable amounts. Free-living fungi are quantitatively of minor importance, and the picoplankton fraction might serve only as a place for the dispersal of zoospores or cysts. However, organisms belonging to *Cryptomycota* were likely underestimated by qPCR due to a primer mismatch selecting against them and therefore might be of larger importance in Lake Stechlin than seen in the qPCR. The pronounced decline of both microbial groups at the end of the experiment might mark the beginning of the death phase due to starvation (pollen grains were completely emptied at that time), grazing, parasitism, viral lysis, or accumulation of toxic metabolites.

**Figure 4:**
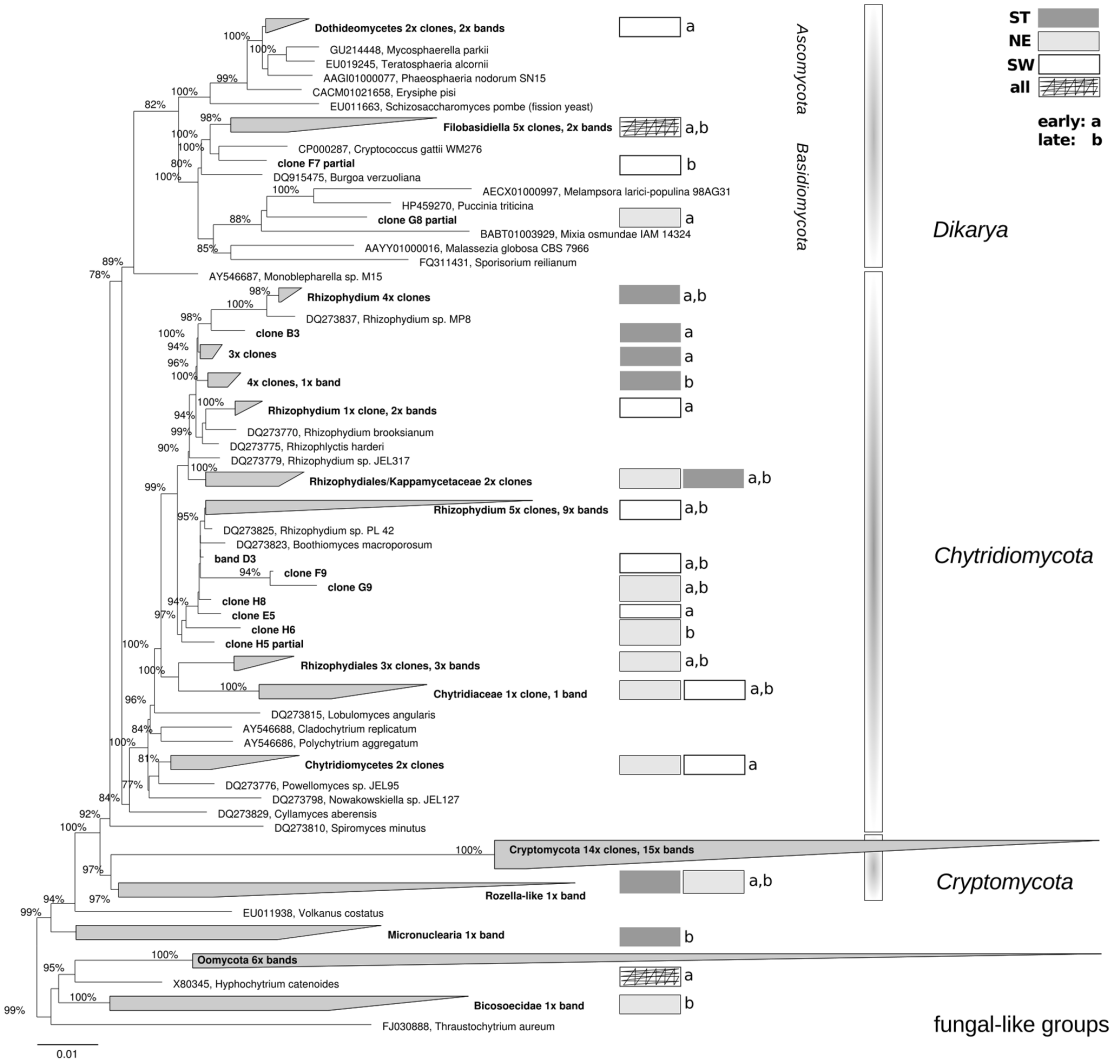
Phylogenetic tree of environmental fungal sequences found during the experiment. Maximum likelihood tree of all fungal and fungal-like ribosomal LSU sequences obtained by clone libraries and DGGE bands of all three treatments during the course of the pollen experiment (bold letters). Only resample values above 75 based on 1000 calculations for all full-length sequences are given. Partial sequences were added to the base tree by parsimony. Additional information for unique clusters of a certain treatment or phase of degradation is indicated. Fungal-like sequences were derived from the primer tests (see Material S1).

Our microscopy studies revealed a variety of basal forms as well as filaments and propagules (conidia) from higher fungi (Figure 2). We aimed to systematically recover these forms through fungal specific environmental DNA sequences based on clone libraries and sequenced DGGE bands of the ribosomal LSU RNA gene. Inasmuch as the sequencing identified many taxa of the most basal fungal lineages and a few related to the two fungal crown phyla of *Dikarya*, we were successful in spanning a broad evolutionary distance across fungal lineages (Figure 4). However, not all visible filamentous fungal forms, such as the aquatic hyphomycete seen in Figure 2, could be recovered by DNA sequencing. In all, we affiliated 92 sequences to four fungal phyla (*Ascomycota, Basidiomycota, Chytridiomycota*, and *Cryptomycota*) and 8 sequences to three fungal-like groups (*Oomycota, Micronuclearia*, and *Bicosoecidae*). *Chytridiomycota* sequences were prevalent (49%), and most of them were clustered into the order *Rhizophydiales*. They were followed by a group of sequences in proximity to *Rozella* sp. (30%), which we affiliated to *Cryptomycota* (Figure 4). These sequences were reduced in the divergent LSU regions D2 and D8, resulting in shorter sequences than other fungal phyla (>100 bp). A small percentage of sequences (13%) were identified as higher fungi (*Dikarya*); most of these sequences belonged to the dimorphic genus *Filobasidiella* (*Basidiomycota*). The remaining 8% of the sequences fell within the fungal-like phyla, and *Oomycetes* was prominent.

In our study, the majority of recovered fungal species belonged to the lower zoosporic fungi (particularly within the order *Rhizophydiales*). Several lake studies demonstrated that this order includes a number of freshwater lineages without any close relatives [12], [67]–[69] and that pine pollen can host at least six species of *Rhizophydiales* [10]. This distribution of fungal phyla is in agreement with our assumption that the small surface area of pollen grains will select for lower fungal phyla. Filamentous fungi established their hyphal network by aggregating multiple pollen grains together and thus substantially increased the substrate surface size (microscopic observations, [20]). [12] used microscopy to describe the similar enrichment of yeasts and zoosporic fungi in surrounding pollen grains and the restriction of filamentous fungi to larger POM. The yeast-like *Cryptococcus* species (yeast form of *Filobasidiella* species) can grow dimorphically (filamentous and yeast-like; [70]), which might have been a major advantage in our incubation experiment. These organism are often found in surface waters as saprobes of insects, plants, or leaf litter and sometimes as mycoparasites or pathogens [71]. Their consistent appearance in aquatic studies (e.g., [15], [72]) is peculiar and certainly deserves further attention. Our findings have implications for studies that include different POM size classes. Ergosterol-based fungal biomass estimations should be complemented by molecular tools or other markers (such as chitin, which is present across most fungal phyla) to account for non-ergosterol-containing fungi and to avoid misinterpretation, especially when using FPOM.

**Figure 5:**
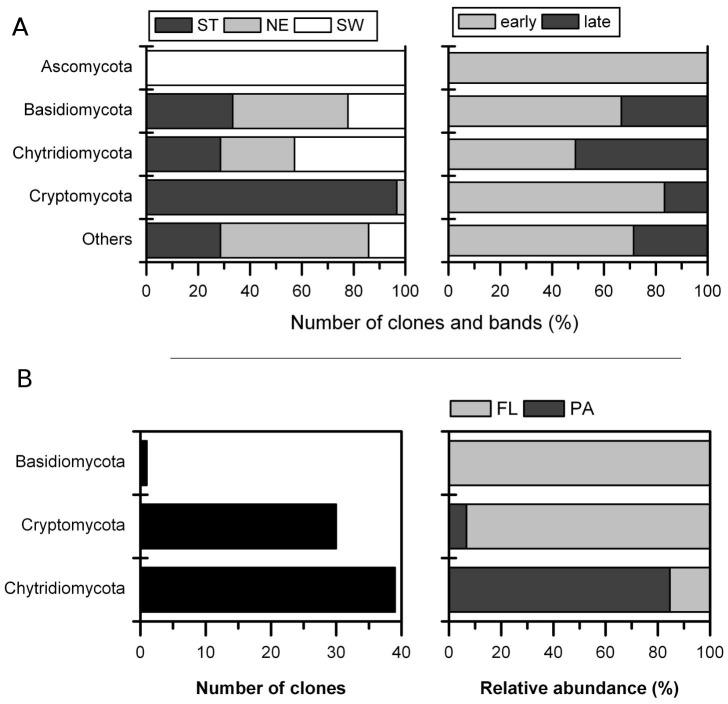
Distribution of fungal taxa across treatments and size fractions. Summary of recovered sequences. (**A**) Compiled data from Figure? 3 summarizes the distribution of fungal phyla and their occurrence during the experiment across treatments. (**B**) Summarizes the phylogenetic distribution of clones obtained for size-fraction analysis of Lake Stechlin treatment (ST) and the corresponding origin (FL or PA).

Furthermore, we studied the fungal phyla distribution between the three lake systems and their occurrences during the incubation period (Figure 5A). With respect to lake type, *Ascomycota* were present only in SW treatments, and *Cryptomycota* occurred almost exclusively in ST treatments. The other phyla were not associated with a particular lake type, although *Chytridiomycota* seemed to have lake-specific lineages (Figure 4). During the experiment, more than 80% of *Ascomycota* and *Cryptomycota* were recorded in the early phase of the experiment (Figure 5A). The low number of ascomyceteous *Dothideomycetes* may derive from the littoral vegetation in the lakes [73] and thus may have been introduced from lake water or pollen grains. The high frequency of sequences belonging to *Cryptomycota* was surprising, since they usually occur at a very low frequency in freshwater systems (e.g., [74]). *Cryptomycota* represent a group of newly discovered fungi that have become a focus of current mycological research and were proposed only recently as a new phylum [75], [76]. They were first discovered as purely environmental DNA sequences in an algal decomposition process in 1999 [77]. Since then, *Cryptomycota* were repeatedly recovered in several types of different habitats ranging from marine to terrestrial systems, with freshwater systems being most prevalent [78]. [79] suggested that members of *Cryptomycota* might be phytoplankton parasites and therefore have a similar niche as their *Chytridiomycota* relatives, while [75] suggested an intracellular parasitic lifestyle that could explain the long branches repeatedly found in phylogenetic trees by rapid evolution. The previously described full-length clone libraries do not discriminate between FL and PA fungi. To better account for the nature of *Cryptomycota*, we investigated fungal diversity of the ST treatment with two additional clone libraries of the PA and FL fractions at day 5 of incubation (39 clones each). *Cryptomycota* were represented by 30 clones, and 93.3% of them belonged to the FL fraction (Figure 5B). If they had a life cycle similar to *Chytridiomycota* (84.6% were PA), they should demonstrate a similar apportionment. These results clearly show a free-living (possibly yeast-like) saprotrophic life for the retrieved *Cryptomycota* cluster from Lake Stechlin. *Basidiomycota* were also detected in the FL fraction which may point to a yeast-like growth, however, they were only represented by a single clone.

In contrast to fungal organisms, we roughly delineated the occurring bacteria by obtaining 43 sequences derived from DGGE bands (data not shown). We found *Betaproteobacteria* to be most prevalent (53%); this bacteria frequently colonizes organic aggregates such as lake snow [80] and is often closely associated with freshwater algal species [81]. *Betaproteobacteria* are among the most widely distributed phyla in freshwater systems [82]. Additionally, *Betaproteobacteria* are known to grow preferentially on polymeric organic matter [83], [84], which suggests a presumably important role in pollen degradation. The remaining sequences matched *Actinobacteria* (12%), *Alphaproteobacteria* (9%), *Bacteroidetes* (5%), *Firmicutes* (2%), and *Cyanobacteria* (2%). The retrieved chloroplast sequences (17%) belonged to *Chrysophyceae* and *Pinus sylvestris* (added pollen). We concluded that *Betaproteobacteria* and *Chytridiomycota* were the most successful in our pollen incubations.

### Lake-specific source communities trigger pollen degradation

**Figure 6:**
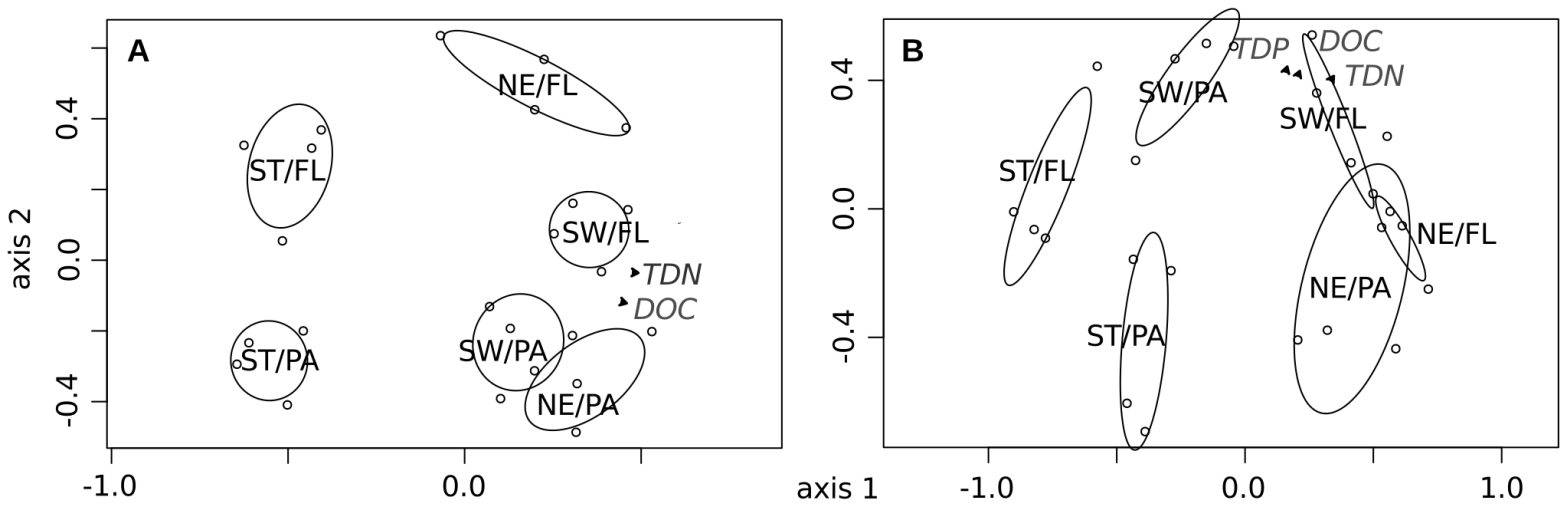
Fungal and bacterial community ecology analysis based on molecular fingerprints. NMDS plots based on distance matrices of bacterial (A) and fungal (B) DGGE patterns. Ellipses were based on standard deviations around the centroids of the corresponding categories (treatments/size fraction). Non-overlapping ellipses can be considered significantly separated groups (Oksanen et al., 2011). Significant environmental variables were plotted as arrowheads into the NMDS plots. (Stress values: Bacteria  = 0.21, Fungi  = 0.19).

The occurrence of *Cryptomycota* pointed to a strong difference in fungal communities introduced by habitat water from the different lakes. DGGE analysis revealed a precise overview on the temporal dynamics of both microorganism groups during the incubation. NMDS analyses of the DGGE fingerprints from fungi and bacteria clearly reflected the different lake types (Figure 6; significant separation of centroids, envfit: p<0.001). Communities of both size fractions formed distinct groups within each lake type (Figure 6). Furthermore, both microbial communities of the NE and SW treatments were more similar to each other than to ST communities. The three lakes do harbor lake-specific bacterial communities [31] and for bacteria it is known that they are the more indigenous the longer the water residence time of a lake [85]. This might explain the strong separation of ST communities (>60 years residence time) and the more similar communities found at Lake Fuchskuhle (<10 years residence time). For saprotrophic fungi the concept of lake specificity is not ambiguously described so far [12], [86], but our results would clearly support this concept, also for fungi. In addition, the observed lake-specificity is not lost by adding a uniform substrate. In this context, [87] found that different source communities do not necessarily converge on the same substrate. Moreover, the convergence of microbial communities decreased after increasing the nutritional quality of the substrate and aligning the greater phylogenetic distance of microbial communities. As a high-quality substrate, pollen exhibited the same effects in our experiment. In addition to this substrate quality effect, a certain historical pre-adaptation of the microbial community to a known substrate can be expected [87]. Since we sampled our lakes at the end of the pine pollination season (16th of June), microbial communities most likely had been adapted to this substrate for weeks. Therefore, pollen addition to the pre-adapted, lake-specific source community does not necessarily exhibit a further strong selection pressure. Distinct size fractions within these lake-specific communities point to a fungal and bacterial sub-community that is specialized to only one of the fractions. For bacteria, we have already found several examples, e.g., free-living freshwater lineages of *Actinobacteria* [31], [88]. Interestingly, the structure of the fungal community according to size fractions seems not to be universal, as it was not possible to distinguish the FL from the PA sub-communities of the NE treatment. Apparently, the occurrence of a distinct fungal FL sub-community is lake specific.

### The environment of the fungal bacterial niche overlap

Despite the similarities between fungal and bacterial communities seen in Figure 6, they were statistically weakly correlated to each other (Mantel test: r = 0.322; p<0.001). However, when comparing bacterial with fungal communities within each size fraction, correlations were more pronounced, especially for the FL fraction (Mantel test: PA: r = 0.375, p<0.01; FL: r = 0.603, p<0.001). Throughout the treatments, DOC and TDN seem to fit well to the given community structure and TDP was only found to fit the fungal community structure (Figure 6). In addition, the initial pH (categorical, not shown) described the community differences between treatments well (highly correlated with DOC and TDN, rho = 0.94). These parameters were the most prominent factors in discerning the communities of the three systems. These findings seemed to be consistent with previous results for bacteria. [89] ranked pH as being most strongly correlated to freshwater stream bacterial communities while DOC and nitrogen were contributing factors. A strong structuring effect of pH was also found for bacterial community composition in lakes [42], [31], [90] and was affirmed as the sole factor at the continental scale for soil bacteria [48]. In addition, humic matter (often not independent of pH) likely played a role in structuring the bacterial community [42], [91]. For fungi, pH was found to have only a weak or no effect on fungal communities, although single phylogenetic groups were clearly influenced by it [47], [49]. Alternatively, [47] found a good correlation of C∶N followed by phosphorous and nitrogen content with specific fungal communities. We also identified phosphorous (TDP) as being uniquely associated to fungal communities. As previously mentioned, the N∶P supply ratio influences the growth of bacteria and fungi differently [39], and dissolved P is negatively correlated with fungal biomass [92] and may also influence the fungal community structure.

In studies on leaf litter decomposition (e.g., [39]) it has been shown that bacteria and fungi compete for the same substrate. Fungi have the capability to actively penetrate into the leaf litter and thus mechanically disrupt dead cells of the leaf litter. Bacteria follow the fungal colonization of leaf litter from the inside after fungal colonization [91]. It is very likely that a similar succession of fungi and bacteria also occur on pollen. However, numbers of both particle-associated bacteria and fungi (Figure 3) rather point to a simultaneous development of both microbial groups which may have been the result of the relative low temporal resolution of our sampling.

### The B∶F ratio is not only driven by the substrate quality in aquatic realms

By comparing the three different treatments, we discovered systematic differences in bacterial and fungal dynamics. The community composition and the B∶F ratio varied between experimental phases and lake types (Table 3; significant differences between treatments K.–W. test, H(2) = 6.092, p = 0.048). These differences are mostly driven by changes in fungal ribosomal gene numbers, while absolute bacterial gene numbers (FL+PA) stayed rather constant (Figure S3). Thus, if we assume an exploitation competition between bacteria and fungi, something other than bacterial biomass had to drive these shifts in fungal biomass between the treatments. As mentioned above no significant differences in pollen decomposition and nutrient release between the lake systems were found [20]. Since pollen provided the same amount of particulate C, N and P in all three treatments, the major differences between the treatments are pH and DOC (dissolved humic matter). These parameters also correlate well with the B∶F ratio, particularly in the late phase of the experiment in which a significant correlation with TOC, DOC, and pH (Spearman's rho  = 0.74, 0.73, 0.73, respectively; p<0.001) occurred. In line with our assumption that C∶N might be important [33] we could also detect a linear correlation with C∶N (Pearson's r = 0.78, p<0.001).

**Table 3:** Average ribosomal B∶F ratio of the experimental incubations.

treatment	ST	NE	SW
B∶F	*22.6*	15.0	3.2
*early phase*	*33.3*	29.3	5.4
*late phase*	*14.2*	9.4	2.0

Ratios of bacteria to fungi (B∶F) calculated from qPCR values over both size-fractions as average sum for Lake Stechlin (ST), and northeast (NE) and southwest (SW) basins of Lake Grosse Fuchskuhle. Data for early (day 2 and 5) and late phase (day 10 and 16) are given separately.

Humic matter can likewise have beneficial and detrimental effects on organisms (e.g., [93]) and has often been found to stimulate bacterial growth [40], [42]. A similarly positive relationship to humic-matter-derived DOC has been found for aquatic fungi containing ergosterol in northern European streams [44]. In addition, this was accompanied by a negative relationship to DON bioavailability for bacteria. To access the nitrogen pool bound in humic matter microbial hydrolysis is required, e.g., via leucine-aminopeptidase [94], which is usually, although not exclusively, associated with bacterial activity [36], [95], [96]. We, however, observed a clearly reduced aminopeptidase activity in the humic-matter-rich and low pH treatment of the SW basin (Figure S2). Therefore, we had two dependent factors in our experiment: pH and humic matter concentrations. In order to keep these factors apart, we decided to reanalyze the environmental dataset of 33 rivers described in [44] with a view towards our findings. For this dataset, we calculated the ratios of bacterial and fungal biomasses (measured by microscopic counts and ergosterol) and analyzed them with LASSO including the ranking forms of dissolved C, N, P, and pH. We confirmed the strong influence (+) of DOC towards fungi found by [44] and found a positive influence of dissolved organic nitrogen (DON) towards bacteria. In addition, DIP and pH were of minor importance for this dataset (Figure S4). The subsequent general linear model for DOC and DON was significantly different from the null model for both terms, which confirmed the overall influence of DOC (−) and DON (+) and explained 26% of the variation in the B∶F ratio (df = 31, p = 0.009). Adding pH and/or dissolved inorganic phosphorous did not significantly improve the model, and they were not significant when treated separately. Thus, we suggest that DOC is one of the major chemical parameters that positively influence fungi, while DON has the opposite effect by shifting the ratio towards bacteria. In the investigated range (5.9–8.7), pH has no direct influence; however, it potentially alters the DON bioavailability by reducing the efficiency of enzymes such as leucine-aminopeptidase, which is one of the key enzymes for DON breakdown. We are aware that the pH effect is likely to be stronger at the edges of its range and may play a large role in our SW system. Further manipulative studies with DOC, DON, and pH gradients are needed to resolve these factors in sufficient detail.

## Conclusions

The high biodiversity of co-occurring heterotrophic microorganisms on allochthonous pollen is remarkable since pollen represents a rather uniform, carbon- and nutrient-rich, microbial substrate. Particles produce an additional spatial arrangement that allows for more ecological niches than simple solutes. In particular, pollen favors a diverse fungal community (including freshwater *Cryptomycota*) to coexist with bacteria on and surrounding pollen particles. Fungal and bacterial saprobes are enriched to a different extent, and this seems to rely greatly on specific environmental factors. Fungi were most prominent in acidic dystrophic water, which represents a highly abundant lake type in temperate and boreal regions. In those regions, pollen input can be substantial. In such lakes, fungi would contribute to a substantial part of the microbial loop with important ecological implications, e.g., by altering the entire food-web by adding cross-trophic links to zooplankton filter-feeders [97]–[100]. Therefore, our results focus on aquatic fungi as primary consumers of FPOM and other POM and unequivocally establish their ecological importance in microbial networks of various freshwater ecosystems, particularly acidic dystrophic lakes.

## Supporting Information

Figure S1 **Cluster analysis of replicates for community analysis.** Examination of replicates for fungal and bacterial DGGE profiles of day 2 (Lake-2) and day 16 (Lake-16). 0.2 marks the free-living and 5.0 marks the particle-associated size fraction. Cluster analysis is based on the presence/absence of DGGE bands (Dice) and was calculated with average distances (UPGMA).
(TIF)

Figure S2 **Leucine-aminopeptidase activity at day 3.** Leucine-aminopeptidase activity of the three treatments after pollen addition. Controls consist of treatment water incubated without pollen for three days, respectively.
(TIF)

Figure S3 **Global bacterial versus fungal variation in copy numbers.** Absolute copy numbers of all data points show no global trend between bacteria and fungi. Size fractions were added.
(TIF)

Figure S4 **LASSO analysis of B∶F ratio calculated from the Baltic Sea catchment dataset.** LASSO of multiple standardized variables that are most deterministic for the B∶F ratio in 33 rivers of the Baltic Sea (Jørgensen and Stepanauskas, 2008; re-analysis).
(TIF)

Table S1 **Chemical parameters.**
(TSV)

Material S1 **Primer evaluation.**
(DOC)

Material S2 **Short and partial sequences.**
(TSV)
