# Supplementary figures and images for: Importance of Saprotrophic Freshwater Fungi for Pollen Degradation
Source: PLoS One. 2014 Apr 14;9(4):e94643. doi: 10.1371/journal.pone.0094643 (PMC3986395; doi:10.1371/journal.pone.0094643)

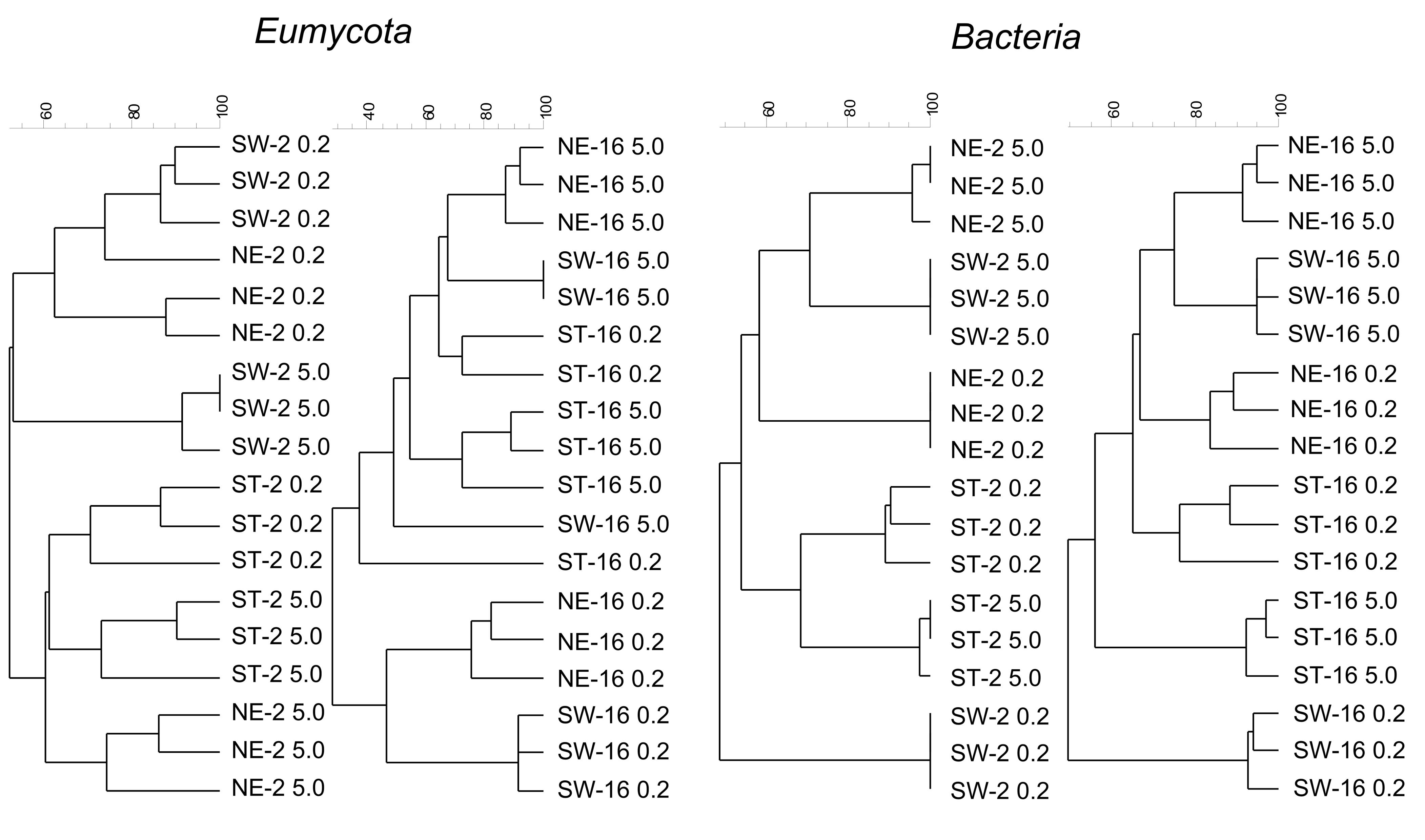

Supplement: Figure S1 — Cluster analysis of replicates for community analysis. Examination of replicates for fungal and bacterial DGGE profiles of day 2 (Lake-2) and day 16 (Lake-16). 0.2 marks the free-living and 5.0 marks the particle-associated size fraction. Cluster analysis is based on the presence/absence of DGGE bands (Dice) and was calculated with average distances (UPGMA). (TIF) [file pone.0094643.s001.tif]

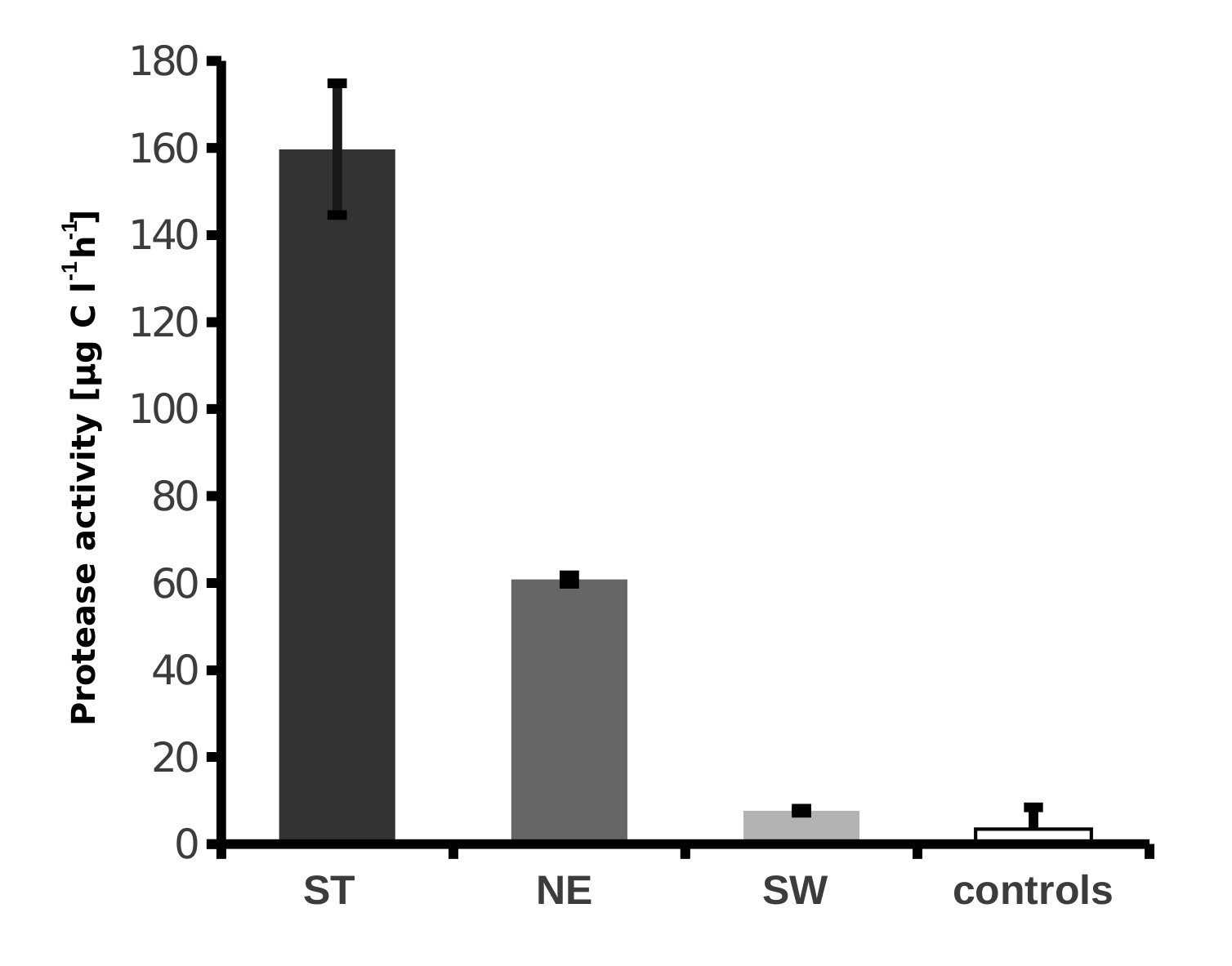

Supplement: Figure S2 — Leucine-aminopeptidase activity at day 3. Leucine-aminopeptidase activity of the three treatments after pollen addition. Controls consist of treatment water incubated without pollen for three days, respectively. (TIF) [file pone.0094643.s002.tif]

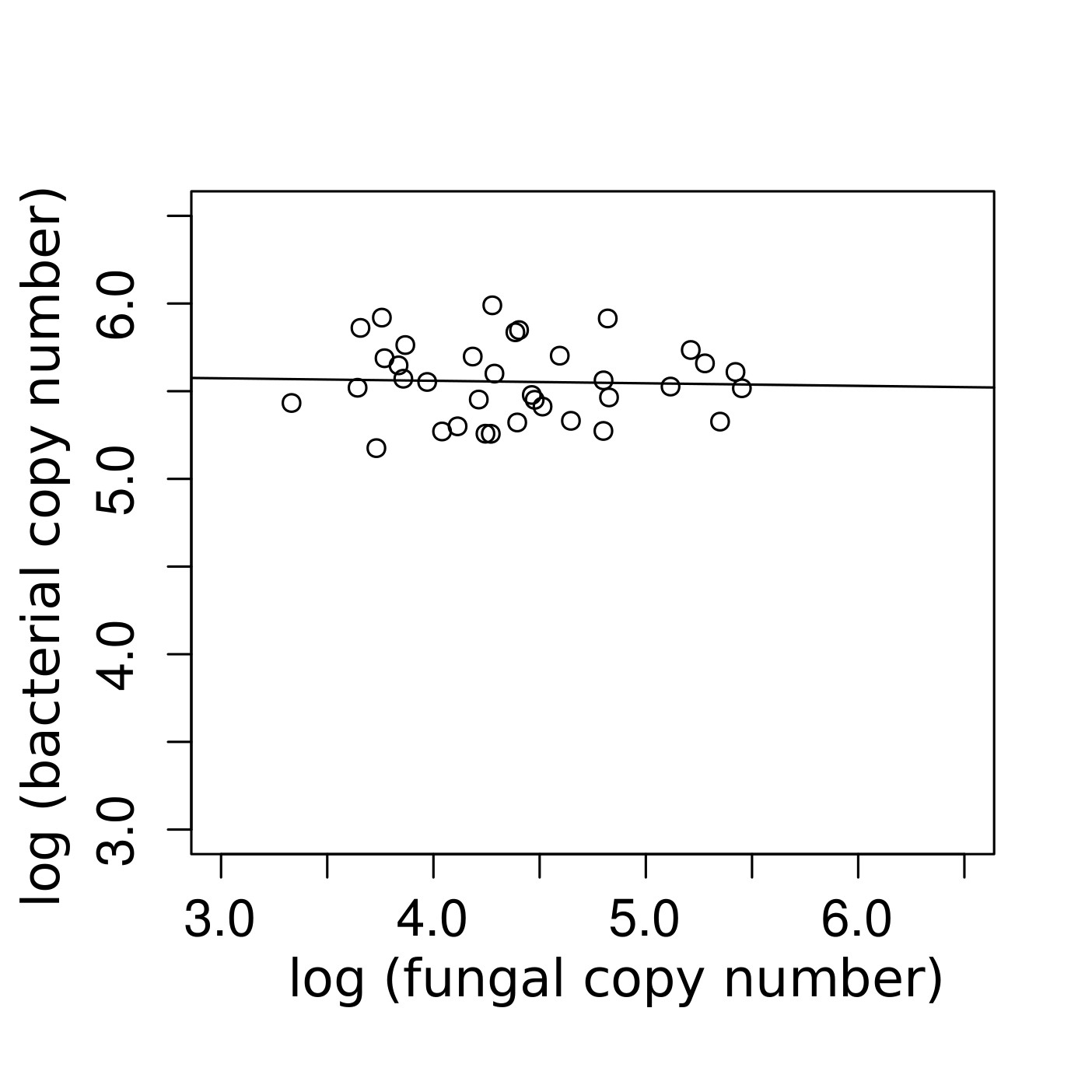

Supplement: Figure S3 — Global bacterial versus fungal variation in copy numbers. Absolute copy numbers of all data points show no global trend between bacteria and fungi. Size fractions were added. (TIF) [file pone.0094643.s003.tif]

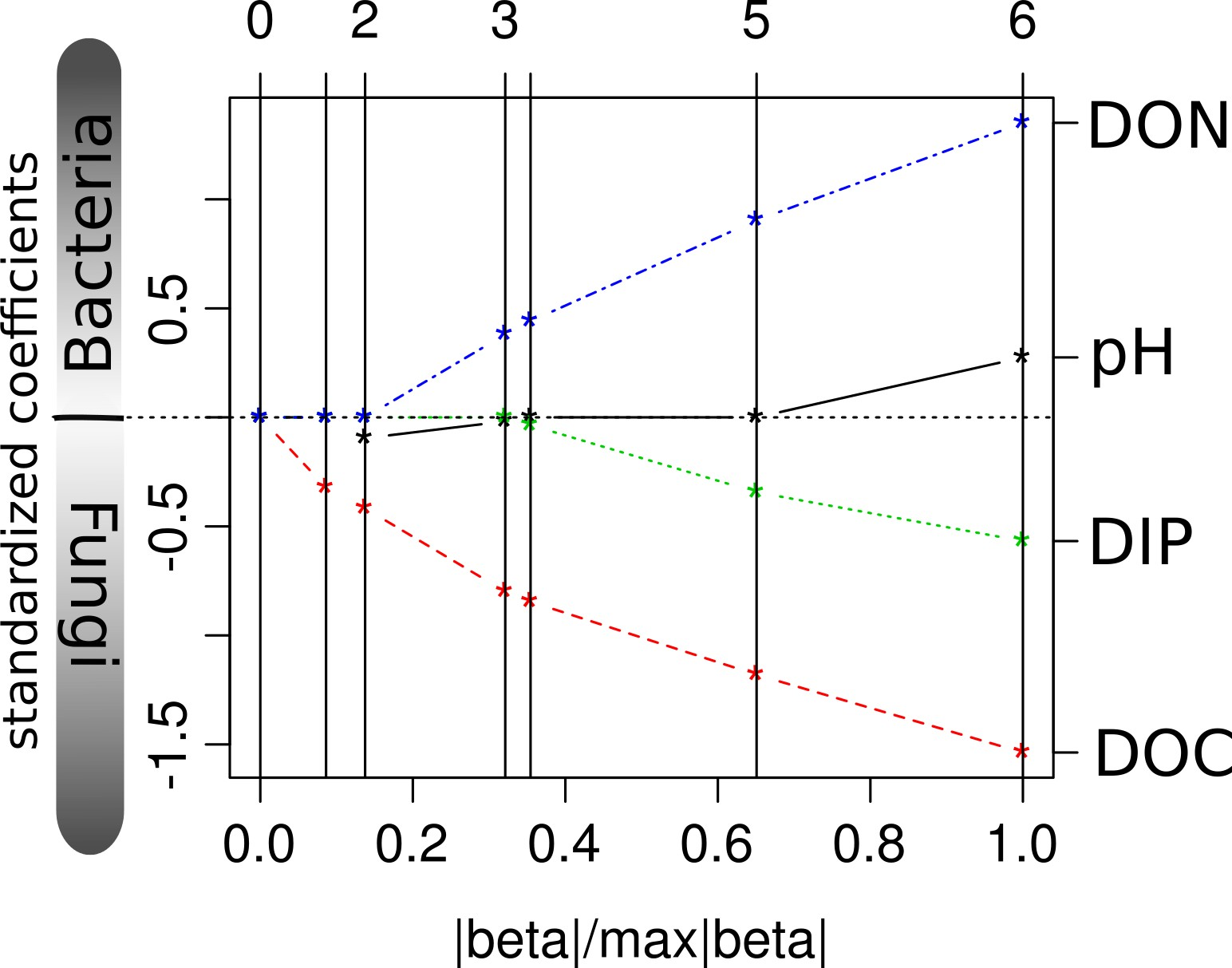

Supplement: Figure S4 — LASSO analysis of B∶F ratio calculated from the Baltic Sea catchment dataset. LASSO of multiple standardized variables that are most deterministic for the B∶F ratio in 33 rivers of the Baltic Sea (Jørgensen and Stepanauskas, 2008; re-analysis). (TIF) [file pone.0094643.s004.tif]
