# Supplementary material for: Importance of Saprotrophic Freshwater Fungi for Pollen Degradation
Source: PLoS One. 2014 Apr 14;9(4):e94643. doi: 10.1371/journal.pone.0094643 (PMC3986395; doi:10.1371/journal.pone.0094643)
Supplement: Material S1 — Primer evaluation. (DOC) [file pone.0094643.s006.doc]

Microbial community composition of treatments was assessed by denaturing gradient gel electrophoresis (DGGE). Since there were merely no experiences with fungal primer sets and planktonic fungi reported in the literature [most of the works are referring to litter degradation studies and/or terrestrial soil, execpt Kagami *et al.* (2012)], we had to evaluate the coverage of fungal groups and false positives for our samples by the corresponding primer sets. For this purpose we tested several established and newly designed primer pairs with our lake water (see all rows with targeted groups: “Fungi” in Table X1). Hereby, primer-pairs were evaluated by (i) their ability to separate bands in the denaturing gradient, which is exemplarily presented in Figure X1, and (ii) to resolve the different fungal lineages (Figure X2). The latter was done by excising, reamplification and sequencing of DGGE bands (see method section, main text). For all primer pairs, we evaluated the phylogenetic affiliation of obtained sequences with BLAST and the quick-add parsimony option of ARB to the LSU base tree (see Phylogenetic analysis, method section) or the SSU base tree (SILVA database, release 108) (Figure X2).

After an initial screening we also evaluated the use of two of the primer-pairs (LR78/RD78, NL1a/LS2) with environmental DNA extracted from water samples during a spring bloom in Lake Stechlin and from sediment-trap material of the adjacent Lake Dagow (Figure X2).

Lower fungi are dispersed among many environmental lineages and unknown fungi, which renders molecular tools ideal to examine their diversity and ecology. Figure X2 shows that caution is needed when interpreting environmental samples analyzed with fungal primer-sets. For example, DGGE primer-pairs ITS3f/ITS4 and NS1/fung are often used in leaf litter decomposition studies (e.g. Nikolcheva *et al.,* 2003; Raviraja *et al.,* 2005; Das *et al.,* 2007), but failed to detect a number of fungi in our study, and even detected a few non-fungal groups. For analysis of samples from our treatments we decided to use primer pair LR78/RD78.

For this primer pair we noticed a certain degree of false positives to zooplankton species (Metazoa) in the environmental DNA samples. However, this was not problematic in our study. For a broader usage of this primer pair in lakes or marine systems, though, we would like to recommend the usage of our competitor oligo RD78-comp (Table X1), which successfully suppresses the parallel amplification of zooplankton DNA when applied as 10 fold primer concentration (not shown).

Additionally to the DGGE primer systems, we evaluated two quantitative real-time PCR (qPCR) systems (Table X1). Therefore, we tested two available primer-sets (Table X1) by using negative controls from common aquatic groups: axenic cultures/DNA of *Daphnia magna* (Crustaceae), *Dictyostelium discoideum (*Amoebozoa*), Microcystis* sp*. (*Cyanobacteria*),* and *Dictyosphaerium* sp*. (*Chlorophyceae*)* as templates*.* PCR conditions for MH2/FungqPCR1 were as described previously (Le Calvez *et al.,* 2009). PCR conditions for QRTfungf/QRTfungr are given in the method section (main text). Standard dilution series proved to be stable over eight orders of magnitudes and had an efficiency of 95.0 ± 3.3%. Of the two primer pairs tested for qPCR, MH2/FungqPCR1 produced false positive signals for most non-fungal templates, except for *D. discoideum* (not shown). Primer-pair RTQfungf/r produced a false positive signal for *D. magna.* When simultaneously analyzing *D. magna* and fungal DNA, fungal quantity was not influenced by the presence of *D. magna* DNA (data not shown). Because we excluded large zooplankton by prefiltration, we employed the latter primer pair. However, we possibly failed to reliably detect *Cryptomycota*, because *Rozella* has two primer mismatches.

**Tables**

**Table X1. List of employed primer-pairs.**

Primer-pairs used in this study for denaturing gradient gel electrophoresis (DGGE) and quantitative PCR (qPCR).

| DGGE primer pair | sequence forward | sequence reverse | annealing temperature | targeted groups | PA [%]/  Gradient [%] | reference |
| --- | --- | --- | --- | --- | --- | --- |
| 341f(GC)/907r | CCTACGGGAGGCAGCAG | CCGTCAATTCMTTTGAGTTT | 55°C | *Bacteria* | *7/40-70* | (Allgaier and Grossart, 2006) |
| ITS3f(GC)/ITS4 | GCATCGATGAAGAACGCAGC | TCCTCCGCTTATTGATATGC | 55°C | *Fungi* | *8/30-80* | *(May et al., 2001)* |
| NS1/fung(GC) | GTAGTCATATGCTTGTCTC | ATTCCCCGTTACCCGTTG | 50°C | *Fungi* | *8/20-55* | *(May et al., 2001)* |
| NL1a/LS2(GC) | AAGCATATCAATAAGCGGAGG | ATTCCCAAACAACTCGACTC | 52°C | *Fungi* | *8/30-50* | NL1a mod. after (Kurtzman and Robnett, 1998) (Cocolin *et al.,* 2000) |
| LR78/RD78(GC) | AGATCTTGGTGGTAGTAGCAA | TGTTTTAATTAGACAGTCAGATTC | 60°C | *Fungi* | *7/30-45* | LR78 mod. after (Vilgalys and Hester, 1990); this study |
| LR7-R/RD1765 (GC) | AGATCTTGGTGGTAGTAGC | GTTTTAATTAGACAGTCAGATTCC | 50°C | *Fungi* | *8(7)/30-45* | LR78 mod. after (Vilgalys and Hester, 1990); this study |
| RD78-comp | CTTTGTTTTAATTAGACAGTCGG-PHO | | 60°C | Zooplankton |  | this study |
| **RTqPCR Primer** | | | | | | |
| MH2/FungqPCR1 | TTCGATGGTAGGATAG | TGTCGGGATTGGGTAATTT | 48°C | *Fungi* |  | *(Le Calvez et al., 2009)* |
| QRTfungf/QRTfungr | CCTTAGACTGACAGATTAA | GTTTGATCTCAGTTCGAG | 60°C | *Fungi* |  | this study |

**Figures – Material S1**

**Figure X1. Optical comparison of DGGE run characteristics of tested primer-pairs.**

Comparison of different DGGE primer-systems. Example shows community profiles of Lake Grosse Fuchskuhle (NE basin) for all tested primer pairs (Table S1) in this study. Each lane represents a sampling date.


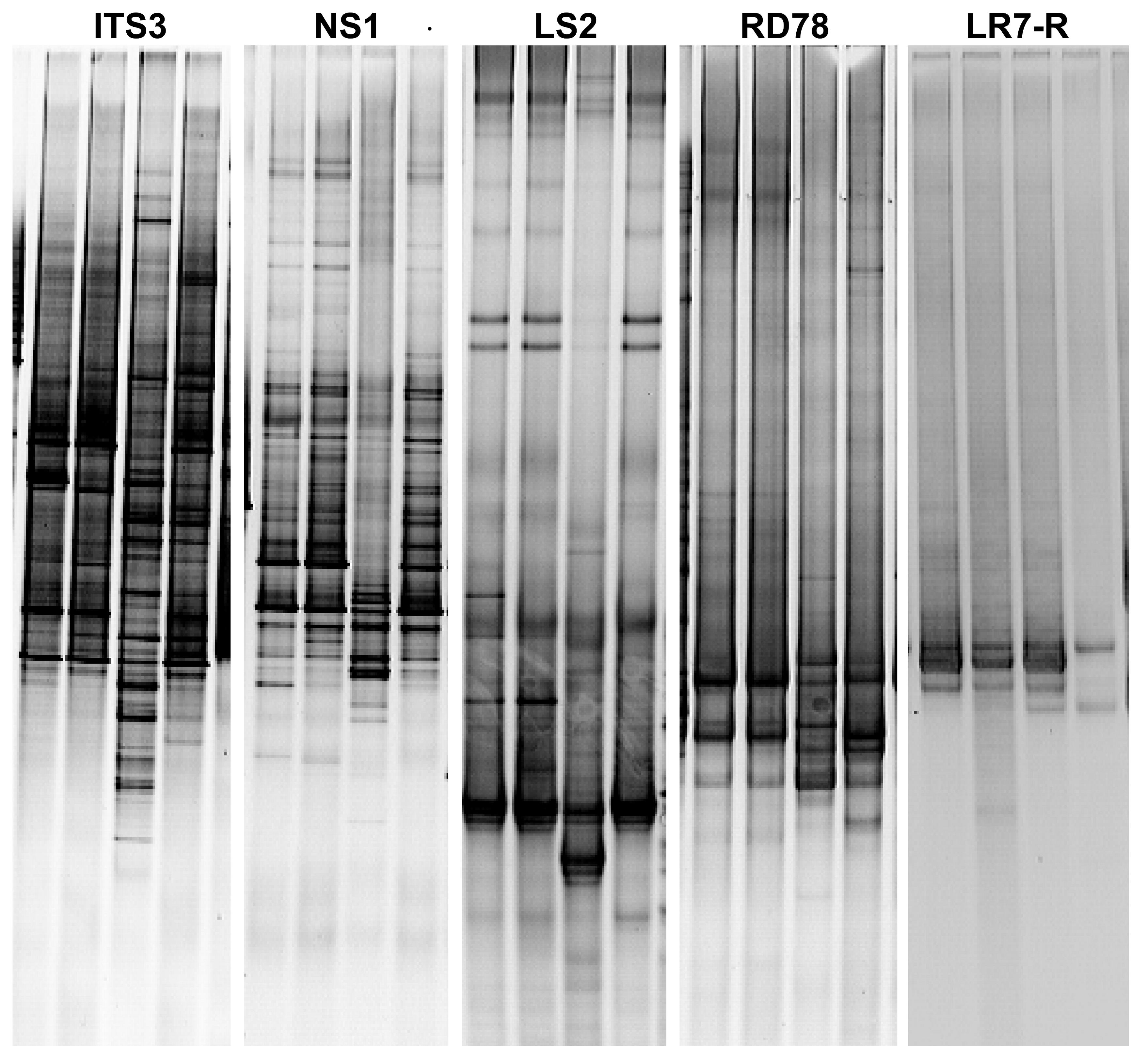


**Figure X2.** **Classification of dominant DGGE bands after sequencing for each primer pair.**

Comparison of different primer pairs by resolving phylogenetic affiliations of sequenced DGGE bands. For two primer pairs (NL1a/LS2 and LR78/RD78) we tested environmental DNA samples obtained during a pelagic spring bloom in oligotrophic Lake Stechlin and from sediment traps in eutrophic Lake Dagow. Color coded blocks mark fungal (-like) groups.

## References

Allgaier M, and Grossart H. (2006). Seasonal dynamics and phylogenetic diversity of free-living and particle-associated bacterial communities in four lakes in northeastern Germany. *Aquatic Microbial Ecology* 45: 115-128.

Cocolin L, Bisson L, and Mills D. (2000). Direct profiling of the yeast dynamics in wine fermentations. *FEMS Microbiology Letters* 189: 81-87.

Das M, Royer TV, and Leff LG. (2007). Diversity of fungi, bacteria, and actinomycetes on leaves decomposing in a stream. *Applied and Environmental Microbiology* 73: 756-67.

Kagami M, Amano Y, and Ishii N. (2012). Community structure of planktonic fungi and the impact of parasitic chytrids on phytoplankton in Lake Inba, Japan. *Microbial Ecology* 63: 358-68.

Kurtzman CP, and Robnett CJ. (1998). Identification and phylogeny of ascomycetous yeasts from analysis of nuclear large subunit (26S) ribosomal DNA partial sequences. *Antonie van Leeuwenhoek* 73: 331-371.

Le Calvez T, Burgaud G, Mahé S, Barbier G, and Vandenkoornhuyse P. (2009). Fungal diversity in deep-sea hydrothermal ecosystems. *Applied and Environmental Microbiology* 75: 6415-21.

May LA, Smiley B, and Schmidt MG. (2001). Comparative denaturing gradient gel electrophoresis analysis of fungal communities associated with whole plant corn silage. *Canadian Journal of Microbiology* 47: 829-841.

Nikolcheva L, Cockshutt AM, and Bärlocher F. (2003). Determining diversity of freshwater fungi on decaying leaves: comparison of traditional and molecular approaches. *Applied and Enviromental Microbiology* 69: 2548-2554.

Raviraja NS, Nikolcheva LG, and Bärlocher F. (2005). Diversity of conidia of aquatic hyphomycetes assessed by microscopy and by DGGE. *Microbial Ecology* 49: 301-7.

Vilgalys R, and Hester M. (1990). Rapid genetic identification and mapping of enzymatically amplified ribosomal DNA from several Cryptococcus species. *Journal of Bacteriology* 172: 4238-4246.
